# Advances in the application of mesenchymal stem cells, exosomes, biomimetic materials, and 3D printing in osteoporosis treatment

**DOI:** 10.1186/s11658-021-00291-8

**Published:** 2021-11-14

**Authors:** Xiao-Yu He, Hai-Ming Yu, Shu Lin, Yi-Zhong Li

**Affiliations:** 1 Department of Orthopaedics, The Second Affiliated Hospital of Fujian Medical University, No. 34 North Zhongshan Road, Quanzhou, 362000 Fujian Province China; 2 Centre of Neurological and Metabolic Research, The Second Affiliated Hospital of Fujian Medical University, No. 34 North Zhongshan Road, Quanzhou, 362000 Fujian Province China; 3 Diabetes and Metabolism Division, Garvan Institute of Medical Research, 384 Victoria Street, Darlinghurst, Sydney, NSW 2010 Australia

**Keywords:** Mesenchymal stem cell, Exosome, Biomimetic materials, 3D printing, Osteoporosis

## Abstract

Owing to an increase in the aging population, osteoporosis has become a severe public health concern, with a high prevalence among the elderly and postmenopausal adults. Osteoporosis-related fracture is a major cause of morbidity and mortality in elderly and postmenopausal adults, posing a considerable socioeconomic burden. However, existing treatments can only slow down the process of osteoporosis, reduce the risk of fractures, and repair fractures locally. Therefore, emerging methods for treating osteoporosis, such as mesenchymal stem cell transplantation, exosome-driving drug delivery systems, biomimetic materials, and 3D printing technology, have received increasing research attention, with significant progress. Mesenchymal stem cells (MSCs) are pluripotent stem cells that can differentiate into different types of functional cells. Exosomes play a key role in regulating cell microenvironments through paracrine mechanisms. Bionic materials and 3D printed scaffolds are beneficial for the reconstruction and repair of osteoporotic bones and osteoporosis-related fractures. Stem cells, exosomes, and biomimetic materials represent emerging technologies for osteoporosis treatment. This review summarizes the latest developments in these three aspects.

## Introduction

Osteoporosis is an age-related bone disease characterized by low bone mineral mass and bone microarchitecture degradation, leading to an increased risk of fragility fractures [1]. Owing to the increasing population of older adults, the healthcare cost and socioeconomic impact associated with osteoporosis are expected to increase [2, 3]. In China, osteoporosis prevalence was 14.94% before 2008 and 27.96% from 2012 to 2015; however, this prevalence has increased significantly in the last 12 years, especially among postmenopausal adults [4]. Metabolic osteopathy can be diagnosed using non-invasive methods, such as X-ray radiation, to detect fracture-prone bones [5–8]. Additionally, X-ray images of fracture-prone bones can be analyzed to determine bone mineral density (BMD). Vertebral compression fracture is the most commonly occurring osteoporosis-related fracture [9]. Although bone cement is used to strengthen the vertebral body during surgery to relieve pain in patients with vertebral body fractures, patients with severe osteoporosis may still exhibit serious surgical complications, such as delayed union, nonunion, and bone cement leakage [10–13].

Osteoporosis diagnosis, fragility fracture prevention, risk assessment of fractures, and the treatment and rehabilitation of fractures have received increasing research attention. Regular physical activity, adequate calcium and vitamin D intake, and regular bone loading play an indispensable role in osteoporosis management [7, 14, 15]. However, there is, presently, no complete treatment for osteoporosis.

Therefore, researchers are currently exploring the application of stem cells, exosomes, biomimetic materials, and 3D printing in treating osteoporosis. This review article collects, analyzes, and summarizes the results of domestic and foreign research in the above-mentioned fields. The aim of this review was to examine the latest research findings, potential applications, and challenges of novel therapies in treating osteoporosis.

## Mesenchymal stem cells

Mesenchymal stem cells (MSCs) are multipotent stromal cells capable of self-renewal and differentiation into mesoderm cells, such as bone, fat, and cartilage cells, and other embryonic lineages [16, 17]. Thus, the use of MSCs is extremely promising for cell therapy in regenerative medicine.

### Sources and classification of stem cells

**Fig. 1:**
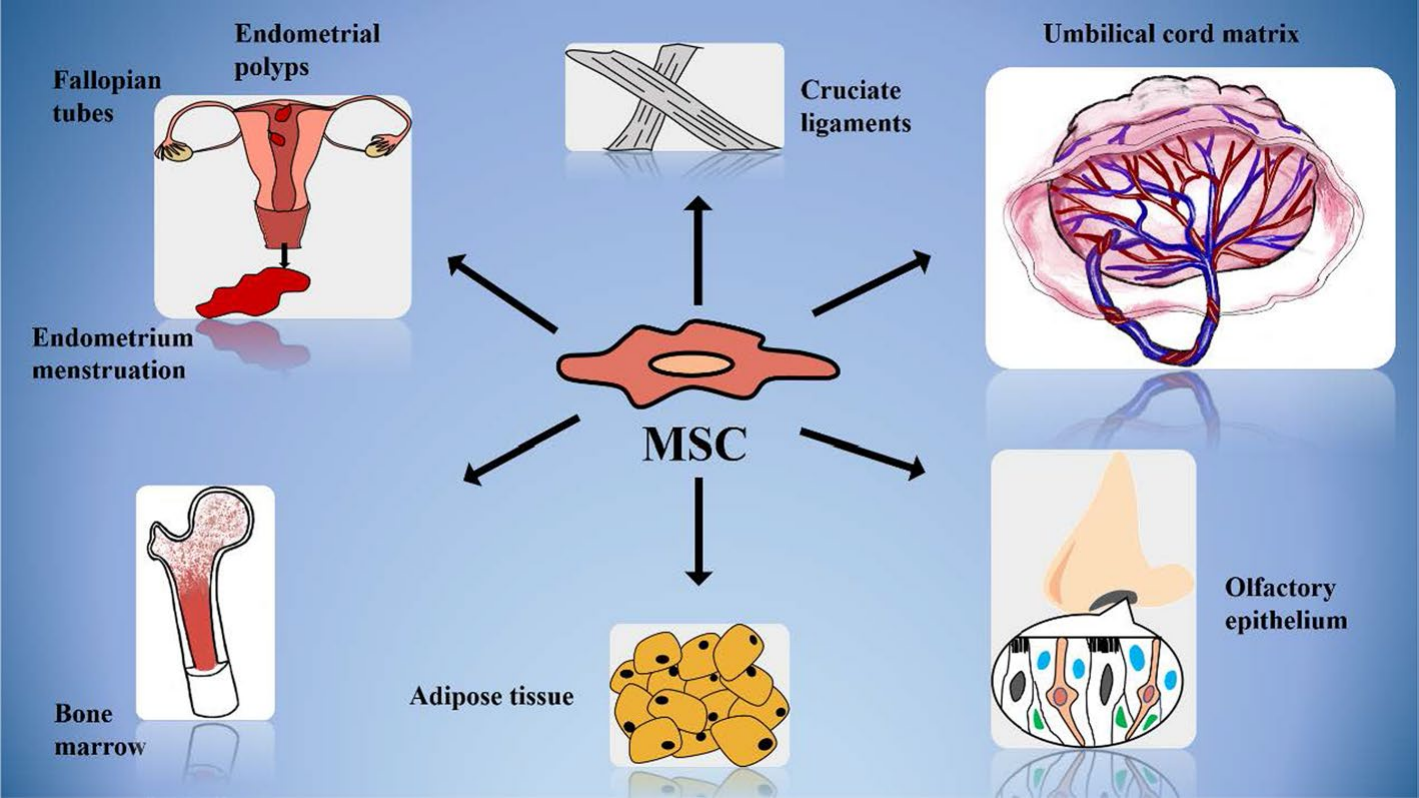
The origin of mesenchymal stem cells (MSCs)

Stem cells are mainly divided into adult stem cells and embryonic stem cells (ESCs). Adult MSCs are widely distributed in various tissues and internal organs in the body, including endometrial menstruation tissue, endometrial polyps, fallopian tubes, bone marrow, cruciate ligament, umbilical cord matrix, adipose tissue, and olfactory epithelium (OE) (Fig. 1) [16–18]. ESCs, which produce three germ layers after directional differentiation in the culture medium, can only be isolated from the inner cell mass of three kinds of embryos (mouse, monkey, and human) at the blastocyst stage [19]. Owing to the shortage of donors, limited number of cells, and ethical considerations, the use of ESCs is largely restricted, and adult stem cells have become the most used stem cells in experiments and clinical trials [20].

### Characteristics and mechanism of stem cells

Most MSCs adhere to plastic support and are easily enriched with serum medium. Moreover, it has been reported that the fractions of MSCs are heterogeneous, with different colony sizes, cell morphologies, and differentiation potentials in fibroblast colony-forming unit (CFU-F) assays. Additionally, in vivo administration of MSCs can modulate immune function by inducing peripheral tolerance and migration to injured tissues [17].

MSC-differentiated cells play an essential role in bone formation, including modeling, remodeling, and regeneration [21]. MSCs are concentrated and differentiated into chondrocytes to form cartilage growth plates, which are then replaced by new bones, or they directly differentiate into osteoblasts to generate bone through intramembranous ossification [21].

### Recent discovery and application of MSCs in osteoporosis and fragility fractures

**Fig. 2:**
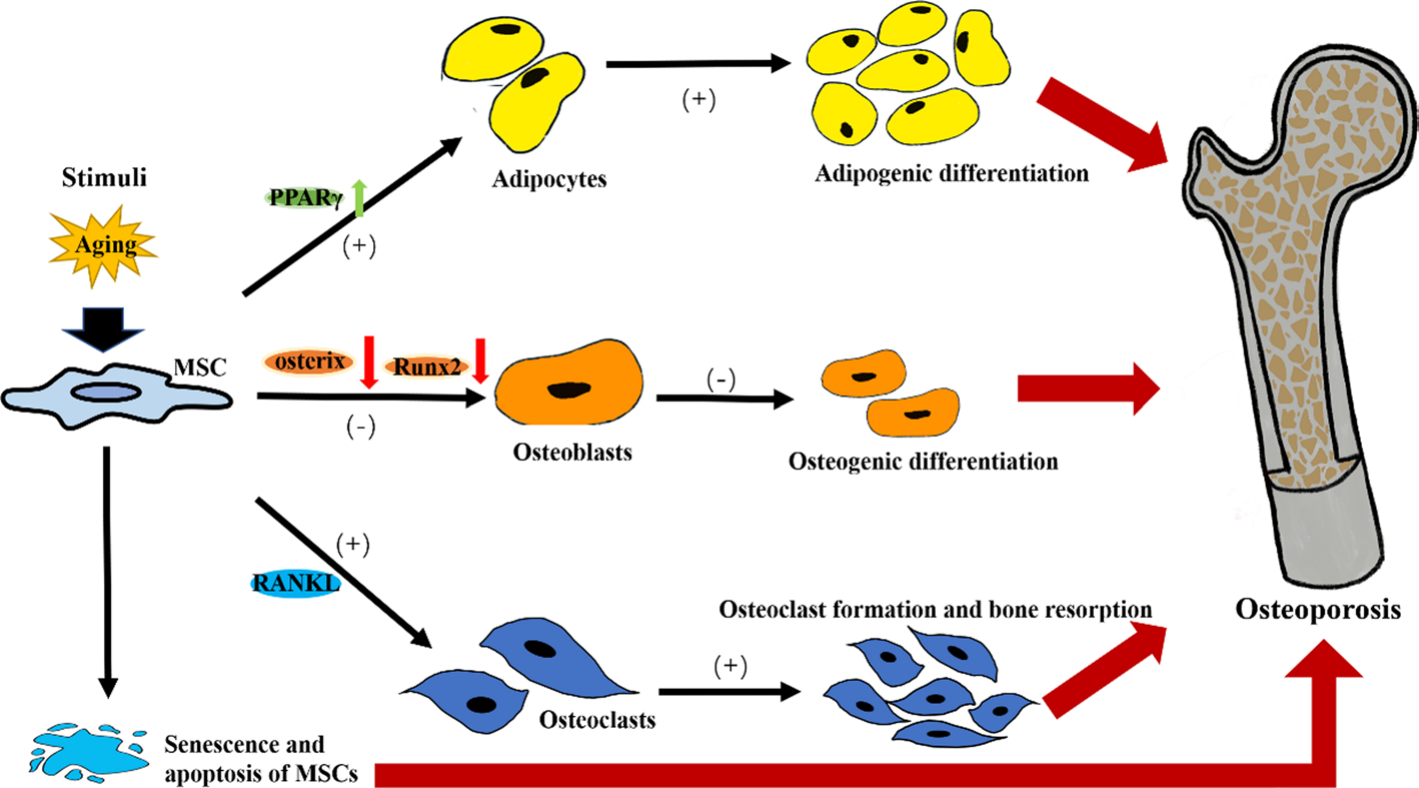
The role of MSCs in the pathogenesis of osteoporosis. It was found that aged MSCs have increased expression of PPARγ and decreased expression of Runx2 and osterix. PPARγ, as an adipocyte-specific transcription factor, inhibited osteoblast development and accelerated adipocyte differentiation. Meanwhile, the reduction of Runx2 and osterix expression, working as osteoblast-specific transcription factors, also negatively regulated bone formation to a certain extent. In addition, the expression of RANKL, which induces osteoclast formation and promotes bone resorption, also contributes to breaking the balance between bone formation and resorption and leads to osteoporosis

MSCs play a crucial role in both the pathogenesis and therapy of osteoporosis due to their multi-directional differentiation potential and self-renewal ability [22]. On the one hand, internal and external stimuli, especially those related to aging, not only cause partial senescence and apoptosis of MSCs, but also regulate the differentiation direction of the remaining MSCs by disrupting the relative stability of the microenvironment (including the transcription factors, signal pathways, and microRNAs) [2, 23]. The down-regulation of runt-related transcription factor 2 (Runx2) and osterix expression, as well as the up-regulation of peroxisome proliferation-activated receptor γ (PPARγ) expression in MSCs, which are important in regulating osteogenic and adipogenic differentiation, inhibit bone formation and increase adipose accumulation, resulting in osteoporosis (Fig. 2) [2, 24, 25]. In other words, adipose stem cells contribute to the precipitation of osteoporosis. In both physiological and pathological conditions, the receptor activator of the NF-κB ligand (RANKL) from bone marrow adipose lineage cells induces osteoclast formation and promotes bone resorption [26]. Due to this reduced osteoblast number and activity, the bone cannot repair and regenerate itself after fractures [27].

On the other hand, studies have shown that direct implantation or intravenous injection of MSCs amplified in vitro can significantly improve bone repair ability by restoring the impaired ability of osteogenic differentiation, increasing bone density, and inhibiting deterioration due to osteoporosis [22, 23]. Several clinical trials have examined the efficacy of bone marrow MSC (BMMSC) implantation and injection in treating osteoporosis using animal models. Adipose-derived stem cells (ADSCs) have also been shown to prevent bone loss, upgrade trabecular bone quality, and increase the expression of molecular markers related to bone turnover in ovariectomy-induced, age-related, and other osteoporotic models [2].Therefore, research attention has focused on developing effective and low-cost therapies to improve osteoporosis treatment outcomes (Table 1).

**Table 1:** Application of MSCs from various sources and their therapeutic outcomes

Source	Characteristics	Administration route	Therapeutic outcomes	References
Adipose‐derived MSCs (ADSCs)	Easy access, adequate source, and high proliferation	Intratibial injection	Prevention of bone loss, upgradation of trabecular bone quality, and increase in expression of molecular markers related to bone turnover	[2]
		Partial transplantation (encapsulated via calcium alginate gel)	Stimulated proliferation, promoted osteogenic differentiation, and enhanced bone regeneration	[28]
Bone marrow MSCs (BMMSCs)	Easy accessibility and high differentiation potential	Partial transplantation	Increased trabecular thickness, improved newly formed osteoids with microstructures, and increased bone stiffness	[2]
		Partial injection	Increased bone mass, reduced rate of bone loss, and osteoporosis prevention	
		Systemic infusion	Prevention of bone loss and strength reduction	
Dental pulp stem cells (DPSCs)	Capability of mediating tissue regeneration and osteogenic differentiation	Systemic infusion (modified by hepatocyte growth factor (HGF))	Strengthened osteogenic differentiation capacities and increased expression of osteogenic-related genes	[29, 30]
Umbilical cord MSCs (UCMSCs)	High osteogenic and proliferative capacity	Partial injection	Increased osteogenic differentiation, increased trabecular bone formation, and reduced bone loss	[31, 32]

### Prospects and research gaps

Several clinical trials and animal experiments have confirmed the positive effects of MSCs in the repair of damaged tissues in various degenerative diseases [2, 20]. However, the precise regulatory mechanism and molecular markers for assessing MSC migration to the bone surface, which is key for bone formation and fracture healing, remain unclear, making it difficult to regulate the activities of MSCs during osteoporosis and fracture treatment [21]. Therefore, future studies should examine the regulatory mechanisms of MSCs in osteoporosis treatment. Additionally, research should focus on the efficacy of autologous adipose stem cells, which are readily available and highly biocompatible, in treating osteoporosis.

## Exosomes

Extracellular vesicles are mainly divided into apoptotic bodies, microvesicles, and exosomes [33]. In 1983, Johnstone et al. first discovered exosomes in sheep reticulocytes [34]. Lipid bilayer vesicles are between 30 and 150 nm in diameter and contain several functional molecules, such as proteins, mRNA, miRNA, and lipids [35]. Exosomes can be secreted by most cell types, including MSCs, immune cells, platelets, cancer cells, sperm, osteoblasts, osteoclasts, and their precursors. They are naturally found in various body fluids, such as urine, blood, amniotic fluid, saliva, semen, and breast milk [33, 35–37]. Owing to their different origins, exosomes carry cell-specific cargoes according to their parent cells [38].

### Exosome formation

**Fig. 3:**
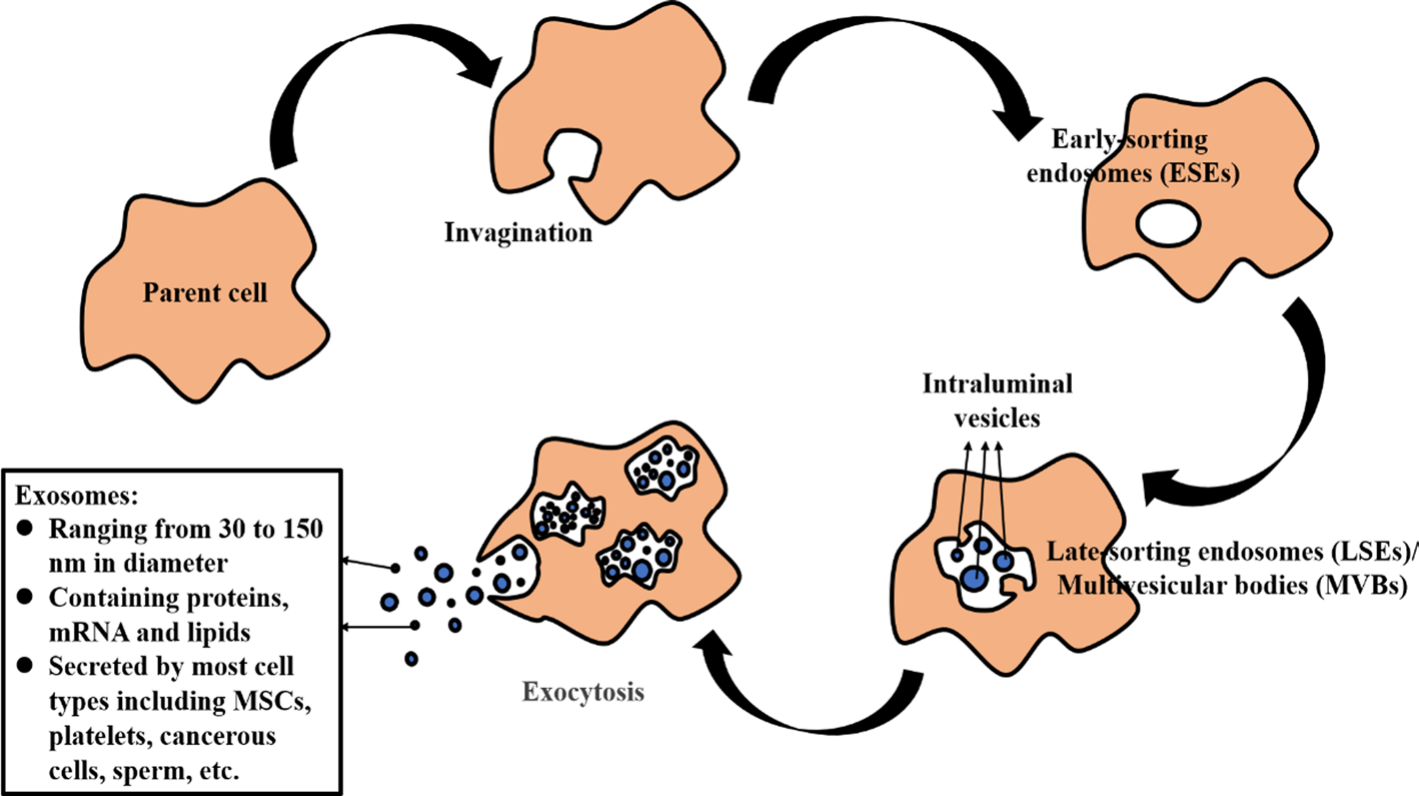
The formation of exosomes

Exosome biogenesis through the endocytosis-exocytosis pathway is divided into three stages. In the first stage, cells with clathrin-coated microdomains on their plasma membranes undergo plasma membrane invagination to form early sorting endosomes (ESEs) [39]. They then diffuse into late-sorting endosomes (LSEs), also known as multivesicular bodies (MVBs), which contain intraluminal vesicles (ILVs) [35]. The last stage is the fusion of multivesicular bodies (MVBs) with lysosomes or plasma membranes, with the release of ILVs into the extracellular environment (Fig. 3) [33, 40].

### Isolation and purification of exosomes

The techniques for isolating exosomes from biological fluids differ depending on the exosome source and concentration [41]. These include ultracentrifugation, polymeric precipitation, immunoaffinity capture (IAC), size exclusion chromatography, filtration, and microfluidic techniques [37, 38, 42].

The most reliable and recognized method in practice is ultracentrifugation. However, this method is costly and time-consuming, prompting researchers to commercialize the polymerization precipitation method. Polymeric precipitation is technically simple and takes less time [33]. Additionally, the IAC method, which only pulls down extracellular vesicles with exosome-specific markers, is helpful for isolating high purity exomes despite its complicated operation [41]. It is more effective than the other methods in isolating exosomes and related proteins (Table 2) [43].

**Table 2:** Comparison of different isolation methods

Isolation method	Advantage	Drawbacks
Ultracentrifugation	Gold standard	High cost, time-consuming
Polymeric precipitation	Easy to operate, short completion time	Low sample volumes
Immunoaffinity capture	Good enrichment, high purity	High operational complexities

### General functions and mechanisms of exosomes

Bone remodeling is the process by which osteoclasts and osteoblasts can replace infected bone tissue with new bone tissue, restore bone mass loss, and repair bone defects caused by activities of daily living and unforeseen events [39]. Bone remodeling involves highly regulated communication and signal transduction between cells. Osteoclasts and osteoblasts coordinate with each other not only in terms of quantity but also spatiotemporally; an imbalance in the bone resorption and bone formation activities of these two cells, respectively, as happens with increasing age and inflammation, can lead to diseases such as osteoporosis [44]. Osteoporosis, one of the most common bone diseases, increases bone fragility and the risk of fracture [39].

Exosomes are widely present in different biological fluids and contribute to communication between cells through specific substances [33]. Additionally, exosomes have been shown to participate in both physiological and pathological processes, including immune responses, homeostasis maintenance, coagulation, inflammation, cancer progression, angiogenesis, and antigen presentation [42, 45].

It has been reported that the abnormal expression of exosomal micro-RNAs (miRNAs) contributes to tissue aging and related diseases, such as osteoporosis. For example, Li et al. reported that the transfer of osteoclast-derived exosomes (miR-214-3p) can inhibit osteoblasts and bone formation [46]. Moreover, studies have shown that exosomal miRNAs, such as lncRNA-MALAT1 in bone endothelial progenitor cell (EPC)-derived exosomes and bone marrow stromal cell (BMSC)-derived exosomes, could enhance bone repair by regulating osteoclast precursors and improving osteoblast activity [47, 48]. It should be noted that different molecules, such as proteins and microRNAs, in exosomes derived from various tissues play essential roles in bone remodeling (Table 3).

**Table 3:** Potential effects of exosome molecules on bone metabolism

Exosomal molecules	Origin of exosomes	Mechanisms	Potential effects	References
miR-214-3p	Osteoclast	Targeting osterix and ATF4 (osteogenic transcriptional factors)	Inhibition of osteogenic differentiation and bone formation	[46]
lncRNA-MALAT1	Endothelial progenitor cell (EPC)	Expressing miR-124 excessively to reverse the migration of bone marrow-derived macrophages and osteoclastic differentiation	Positive recruitment of osteoclast precursors and promotion of their differentiation	[47]
	Bone marrow stromal cell (BMSC)	Mediating miR-34c/SATB2 axis	Enhancement of osteoblast activity	[48]
Protein-Fas	Mesenchymal stem cell (MSC)	Downregulating miR-29b levels to recover Dnmt1-mediated programs	Restoration of the osteogenic differentiation ability of MRL/lpr BMMSCs	[49]
miR-31a-5p	Bone marrow stromal cell (BMSC)	Promoting osteoclast formation and bone resorption	Stimulation of osteoclast differentiation and function	[50]
miR-155	Vascular endothelial cell (EC)	Internalizing vascular EC-secreted exosomes with bone marrow-derived macrophages (BMMs) to inhibit osteoclast activity	Suppression of osteoclast induction	[51]
miR-1192, miR-680, miR-302a, miR-3084-3p, miR-680, miR-677-3p and miR-5100	Mineralizing osteoblasts (MOB)	Targeting Ctnnb1 converging on the β-catenin gene	Promotion of osteogenesis and differentiation of ST2 cells into osteoblast-like cells	[52]
miR-667-3p, miR-6769b-5p, miR-7044-5p, miR-7668-3p and miR-874-3p	Mineralizing osteoblasts (MOB)	Repressing Axin1 to inhibit Wnt/β-catenin signaling		

#### Repair of MSC function

Studies have shown that patients with osteoporosis often suffer from diminished osteogenic differentiation capacity and increased lipid content in the bone marrow tissue. Since MSCs are the homologues of osteoblasts and adipocytes, greater MSC differentiation into adipocytes leaves fewer MSCs for differentiation into osteoblasts [53]. Liu et al. reported that protein-Fas in exosomes reduced expression levels of miR-29b in recipient MRL/lpr BMMSCs after MSC transplantation, thus restoring osteogenic differentiation capability in MRL/lpr mice [49].

#### Decrease of osteoclast activity

Xu et al. reported that high levels of microRNA-31a-5p (miR-31a-5p) in BMMSCs increased osteoclast formation, resulting in age-related bone loss, whereas introduction of antagomiR-31a-5p in the bone marrow microenvironment inhibited mir-31a-5p, thereby reducing osteoclast activity [50]. Additionally, Song et al. found that exosomes secreted by vascular endothelial cells (EC-Exos) had better bone-targeting activity. After absorbing EC-Exos, bone marrow-derived macrophages strongly express miR-155 and inhibit the induction of osteoclasts, thereby treating osteoporosis [51].

#### Promotion of osteoblast differentiation

Cui et al. examined exosomes from mineralizing pre-osteoblastic MC3T3-E1 cells (MOBs) and found high miRNA expression in MOB exosomes and ST2 bone marrow stromal cells. These miRNAs include miR-1192, miR-680, miR-302a, miR-3084-3p, miR-680, miR-677-3p, miR-5100, miR-667-3p, miR-6769b-5p, miR-7044-5p, miR-7668-3p, and miR-874-3p, and could promote the differentiation of ST2 cells into osteoblast-like cells by inhibiting Axin1 and enhancing β-catenin [52]. Similarly, studies have shown that exosome injection can restore the osteoblast differentiation in bones irradiated with 16 Gy for 28 days by increasing calcium deposition and the expression of an osteogenic gene (Runx2) at the molecular level [54].

#### Enhanced immune regulation and inhibition of inflammation

Exosomes derived from BMSCs act as immunomodulatory mediators in cell communication by fusing with T cells and regulating their physiological processes, thereby enhancing the immune mechanism [55]. Exosomes also have a stimulating effect on other immune factors, such as tumor necrosis factor (TNF), natural killer (NK) cells, and dendritic cells (DCs) [33].

#### Promotion of angiogenesis

Qi et al. studied the secretory exosomes of MSCs derived from human induced pluripotent stem cells (hiPSCs, hiPSC-MSC-Exos) in ovariectomized (OVX) rats. They found that hiPSC-MSC-Exos stimulated angiogenesis and bone regeneration in vivo and in vitro [56]. Zhang et al. reached a similar conclusion using a rat model exhibiting femoral nonunion [57].

### Current progress on the clinical use of exosomes

The application prospects of exosomes include, among others, the use of biomarkers, delivery vehicles, drugs, and vaccines in therapeutic interventions [33]. Studies have confirmed that cells in a pathological state increase their EV release rate, as evidenced in cancer cells [58], suggesting that they may play a role in the pathological process of the disease. Therefore, exosomes have been widely used as biomarkers for disease diagnosis. Lu et al. reported that exosomes as a natural carrier system could potentially be used for cardiovascular disease risk assessment and atherosclerosis management [59, 60]. Similarly, exosomes have been used to diagnose HIV infection, Alzheimer’s disease, drug-induced liver injury, and cancer [58, 61–65]. This relatively non-invasive and dynamic monitoring using exosomes has broad prospects in clinical practice [61].

Additionally, exosomes are used as a novel type of nanoscale biopharmaceutical delivery carrier because of the signal exchange function of ligands, nucleic acids, or protein factors attached to the exosomal membrane or wrapped inside the exosomes [66]. Saari et al. used exosomes isolated from LNCaP and PC-3 prostate cancer cells as chemotherapeutic drug carriers to deliver paclitaxel to autologous prostate cancer cells [67]. However, the drug acceptability and targeting ability of naturally secreted exosomes are limited. To improve the specificity and acceptability of delivery exosomes, artificial exosome mimetics made of liposomes or nanoparticles that can accept the required ingredients extracted from natural exosomes have been developed [45]. To improve targeting ability, Alvarez-Erviti et al. used the dendritic cell-derived exosome Lamp2b to target neuron-specific RVG peptides, thereby specifically delivering the relevant RNA drugs to specific brain tissues [68]. Hu et al. also found that the C-X-C motif chemokine receptor 4 (CXCR4)-modified exosomes acquired bone-targeting function. CXCR4-modified exosomes and liposomes that released antagomir-188, which has bone forming and adipogenesis inhibition abilities, were fused to treat osteoporosis [69].

Although the identities and functions of several molecules contained in exosomes are yet to be confirmed, they have been reported to positively affect bone regeneration in animal models. Based on the above findings, it can be concluded that exosomes equipped with active ingredients could represent potential treatment strategies for several skeletal disorders, including osteoporosis and osteoporotic fracture, at the molecular level [38]. Presently, several exosome-based drug delivery systems are under development for disease therapy and are awaiting clinical trials. Moreover, there are several other uses of exosomes in disease treatment that require further research.

## Summary

In summary, exosomes generated without artificial intervention can secrete various molecules and play an important role in treating several skeletal disorders, such as osteoporosis, osteogenesis imperfecta, and fractures. Due to the defects in the expression of the major histocompatibility complex (MHC-I and MHC-II) proteins on the cell surface, exosomes can be used for cell transplantation and therapy [39]. Despite the significant research already conducted on exosomes, many questions regarding the identities, functions, and mechanisms of the molecules present in exosomes remain to be answered.

Although exosomes originate from abundant sources, their real-world application in osteoporosis still requires a considerable amount of research due to the multiple other factors that need to be addressed, including their high cost. Moreover, the processes involved in the utilization of exosomes, such as extraction, identification and purification, require the use of various instruments and specially trained researchers to operate them, and the entire process from isolation to purification is time-consuming and requires tremendous amounts of energy. Furthermore, only targeted exosomes with low purity and small volume may be obtained. Therefore, innovations and developments in extraction and related technologies are essential for the application of exosomes in treating osteoporosis (Table 4).

**Table 4:** Comparison between MSC and exosome transplantation in osteoporosis treatment

Therapies characteristic	MSC transplantation	Exosome transplantation
Sources	Various tissues and organs	Mostly all cell types, including MSCs
Features	Self-renewal ability and multiple differentiation potential	Ability to regulate the microenvironment (transcriptional factors, signaling pathways, etc.)
Immunological rejection	Little	None
Isolation and purification	Easy (high sample volumes)	Costly and time-consuming (low purity)
Operation difficulty	Normal	Complex

## Biomimetic materials and 3D printing

Patients with osteoporosis have a higher incidence of osteoporotic vertebral compression fractures (OVCFs) due to metabolic abnormalities and rapid absorption [70], with a higher prevalence among postmenopausal adults [71]. OVCFs can cause chronic pain and impair mobility, thus severely affecting the quality of life [72]. Existing treatments include conservative therapy and vertebral augmentation (percutaneous vertebroplasty or balloon kyphoplasty). However, confirmation of the effectiveness and safety of percutaneous vertebroplasty and kyphoplasty requires further research, and available evidence does not support the routine use of vertebral augmentation [73, 74]. Therefore, researchers have turned their attention to emerging technologies, such as 3D printing and biomimetic materials.

### Traditional therapy and materials

Conservative treatments for OVCFs include limited bed rest, functional restoration, bracing, physical therapy, analgesics, nerve root blocks, and epidural injections [71, 72]. In patients with chronic persistent pain and restricted movement, vertebral augmentation procedures, including percutaneous vertebroplasty (PVP) and balloon kyphoplasty (BKP), are introduced to relieve intolerable pain and improve motor performance [75–77].

PVP is a minimally invasive method that involves injecting synthetic bone cement into the vertebral body under local anesthesia to strengthen the fractured vertebral body. It is superior to conservative therapy in terms of pain relief [70, 71]. BKP involves the same procedure as PVP but includes the additional step of restoring the original height of the vertebral body using a balloon before bone cement injection, thus reducing the risk of bone cement leakage [78, 79]. Additionally, acrylic bone cement (PMMA) has become one of the most important bone cement components in orthopedic surgery because of its strength and cost advantages. PMMA use involves initial mixing, waiting, working, and hardening [80, 81].

However, available evidence suggests that vertebroplasty is associated with several severe complications during vertebral augmentation, such as bone cement leakage, primarily due to peripheral vertebral wall damage and the dosage of bone cement [10, 74]. Additionally, the use of PMMA has some limitations, including low resistance to high pressure and low adhesion to bone fragments [82]. Another widely used material in orthopedics for fracture and bone repair is titanium and its alloys [83]. Titanium and its alloys, or tantalum (Ta), have excellent mechanical strength and good biocompatibility, making them suitable materials for treating fractures and bone defects. However, the use of titanium alloys in orthopedics is limited by their poor biological activity and osseointegration [84]. Therefore, new technologies and materials for use in orthopedics should be examined.

### Biomimetic materials and 3D printing in osteoporosis treatment

Biomimetic materials are designed and manufactured according to the regulatory functions and biological characteristics of bodily tissue, and they create suitable conditions for restoration and regeneration to promote MSC adhesion, cell differentiation, and tissue repair. Their discovery has encouraged researchers to tentatively apply biomimetic materials in the field of regenerative therapy [85].

Additionally, with the advances in medical imaging, digital information technology, and manufacturing, the application of 3D printing in treating some medical conditions is beginning to receive research attention [86]. 3D printing is a manufacturing technology that deposits layers of materials (metallic materials, non-metallic materials, or medical biological materials) and creates 3D objects based on a digital model of the patient’s anatomical structure, which is obtained through 3D volume rendering [84, 87]. 3D volume rendering is a 3D discretely sampled dataset that is applied to generate 2-dimensional (2D) projections, such as computed tomography (CT) or magnetic resonance (MR) images [87, 88]. Owing to its various advantages, such as shorter operation time, improved efficacy, and simplicity of application, 3D printing is referred to as rapid prototyping, additive manufacturing (AM), or solid free-form fabrication [88]. Since its invention in the late 1980s, researchers have used this technology in various fields, including medicine and surgery [87].

#### Various categories in 3D printing technology

3D printing is divided into different categories based on different manufacturing theories.

Vat photo polymerization is commonly referred to as stereolithography or digital light processing. This technology involves constructing 2D materials by exposing a photo-curable liquid resin to high-intensity light [87, 89]. The entire process requires three key components: an appropriate light source, a vat of light-sensitive resin, and a control system [89]. Although this method is commonly applied in drug delivery and medical device manufacturing, large-scale application in medicine is limited by its complexity, high cost, and relative fragility [87, 90]

Material jetting 3D printing enables the production of parts with accuracy and low material wastage, and involves several steps. First, the photopolymer is jetted onto a build tray and cured with UV light, then the models are soaked in soapy water. Finally, the print is completed after the supports are used to uphold the overhangs, and the model shape is easily removed by melting, hand removal, or pressurized water spraying [89]. The multiport printer allows materials with different hardnesses to be applied in the same print [87]. However, the large-scale application of this technology is not cost-effective.

Binder jet printing is arguably the most successful 3D printing technology in the medical field to date. This technology involves the deposition of color and binder onto a thin layer of powdered particles to construct 3D materials [89]. Post-processing, such as infiltration, is necessary to compensate for low material quantity and binding strength [87]. The 3D materials generated using this technology are highly biodegradable because of the micropore characteristics of the structures, making them suitable implants in treating bone defects [91].

Powder bed fusion (PBF) utilizes a laser or electron beam to melt powders of plastic, nylon, metal, ceramic, or other polymers into 3D objects [87]. Several techniques have been derived from this technology, including selective laser sintering/melting, direct metal laser sintering, electron beam melting, and multi-jet fusion [92]. Other procedures are likely to be similar to those of binder jetting. Owing to the high durability of the finished product, this technology is widely used to manufacture medical devices, such as titanium tantalum (TiTa) alloy products [89, 93].

Material extrusion, known as fused deposition modeling and commonly used among amateurs, handles the unreeled material with a heated extruder of a particular diameter and requires post-processing [87, 89]. The low cost and ease of operation allow the method to be widely used.

Other 3D printing technologies include directed energy deposition and sheet lamination, and several studies are currently being undertaken to improve the existing technologies. The selection of the printing method is based on careful considerations of the advantages and disadvantages (Table 5).

**Table 5:** Various categories in 3D printing technology

Manufacturing theory	Characteristic	Application
Vat photopolymerization	High resolution and printing speed	Drug delivery, medical device manufacturing in orthopedics
Material jetting	Can use different-hardness materials in one print	Medical models
Binder jetting	Coloring	Color coding in anatomy, biodegradable implantable devices
Powder bed fusion	Various derivative techniques, good durability	Medical devices, such as metal and alloyed titanium frameworks in dentistry
Material extrusion	Economical, easy to operate	Most household machines

#### Application of 3D printing in medical practice

3D printing and biomimetic materials have the potential to revolutionize the entire process of orthopedic disease diagnosis and treatment.

**Fig. 4:**
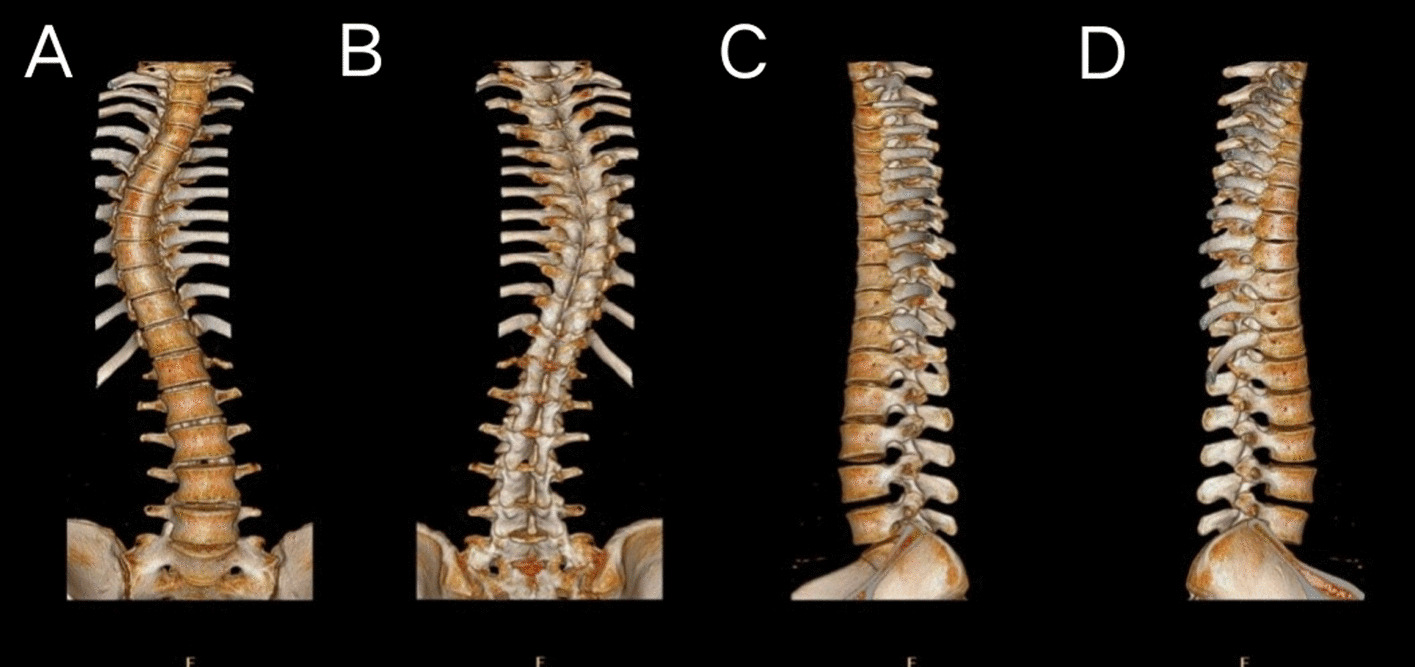
3D imaging of an 18-year-old patient with idiopathic scoliosis generated by processing CT images. **A** Anterior view **B** Posterior view **C** Left lateral view **D** Right lateral view

Before surgical planning, it is essential for surgeons to clearly understand the related anatomy in detail. However, disease-based congenital mutations and structural distortions make it difficult for medical practitioners to reach a conclusion in certain diseases [84, 86]. With the advances in imaging technologies, such as CT and MRI scanning technologies, 3D models of the patient’s specific anatomy can be created using a printer (Fig. 4) [94]. Accurate 3D models of organs can greatly improve the understanding of human anatomy and the outcomes of surgery; additionally, 3D models are widely used in surgical training and patient education. Overall, 3D anatomy models can shorten surgical durations and improve surgical outcomes [84, 88].

**Fig. 5:**
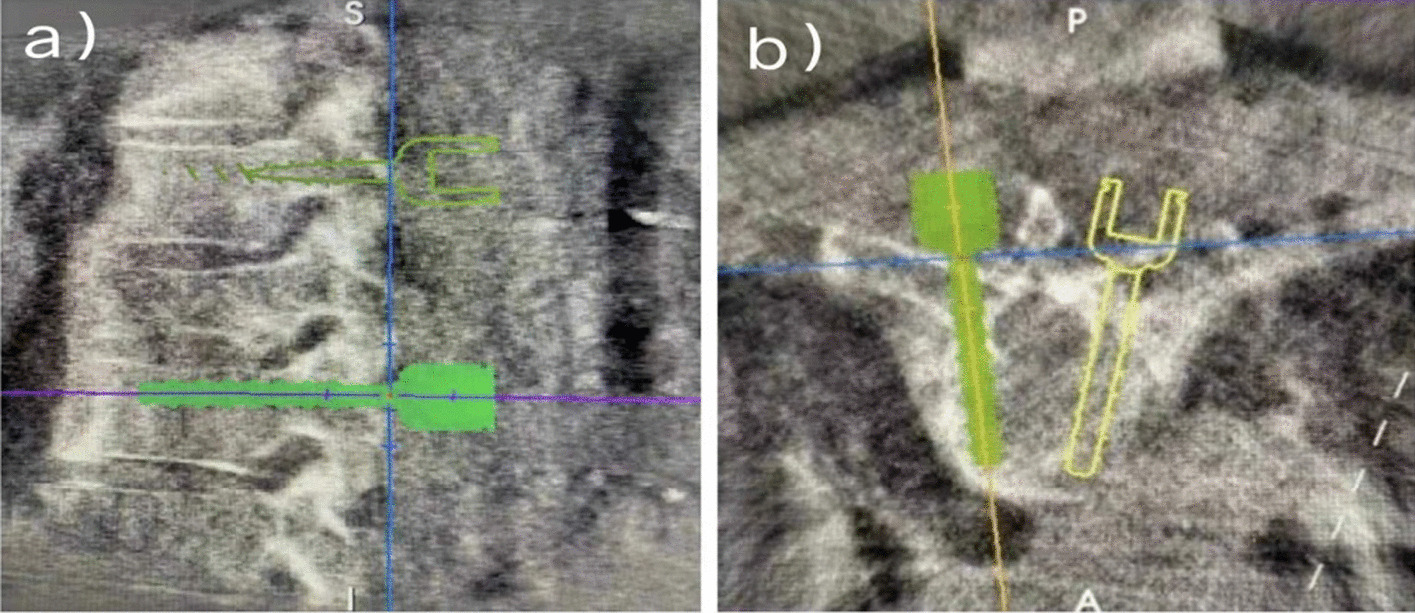
The navigation template of vertebral pedicle screws in an intraoperative three-dimensional image of the spine **a** The sagittal plane which indicates the relative locations of simulative screws in two adjacent vertebrae **b** The transverse plane which simulates the approach and depth of screws in the same vertebra

In addition, 3D printing templates play a key role in orthopedic internal fixation surgery, especially in the placement of pedicle screws during spinal surgery [95]. For doctors, the use of patient-specific 3D models in the screw placement process can significantly simplify and improve the accuracy of surgery (Fig. 5) [95].

Though expensive, the use of patient-specific instruments and customized implants in surgical procedures is associated with improved treatment accuracy and surgical outcomes [84]. Compared with the standard-sized implants used in traditional treatment, individually designed implants can perfectly match the patient’s anatomy [84]. Additionally, some of the printed tissue features can be further strengthened. For instance, Professor Markus Buehler reported that 3D printed bones made of specific biological compounds were 22 times more resistant to fracture and external injury [94]. The high treatment accuracy of patient-specific instruments and custom implants can significantly improve the recovery process [88].

#### Progress of biomimetic materials in the treatment of bone repair

Osteoporosis can cause several bone defects and injuries, such as OVCFs. Traditional treatment with standardized titanium (Ti) alloy implants sometimes fails because of low bioactivity and individual specificity [83]. The use of biological grafts, especially autografts, is the optimal treatment method and the gold standard for fracture management at present. These grafts can not only function as osteoconductive scaffolds, but can also act as a source of osteogenic cells and osteoinductive growth factors [96]. However, their associated drawbacks, including donor-site complications, limited graft quantity, and chronic pain, make surgery using grafts only partially successful [97]. Similarly, the possibility of infection, rejection, disease transmission, and limited osseointegration makes the use of allografts an insufficient option for fracture treatment [96, 98, 99]. Thus, researchers are committed to developing new biomimetic materials that address these disadvantages.

The raw and processed materials in biomaterials cover a large range, from natural polymers to inorganic materials to synthetic polymers and composites [97]. The differentiation promotion function of biomaterials can be activated by immobilizing ECM proteins and peptides (e.g., collagen, fibronectin, osteopontin, and bone sialoprotein) on surfaces [97, 100], which has been shown to have positive effects on cell attachment and osteogenic differentiation [101–103]. For instance, collagen, which forms interstitial structures for hydroxyapatite crystal deposition, can optimize the mechanical properties of bones, including their tensile and compressive strength [97, 104]. In addition, the interaction between proteins and peptides in the ECM also contributes to regulating the growth factors and hormones that play crucial roles in cellular differentiation and bone remodeling [96, 100].

Since the issues related to structural support and fixation, availability, biocompatibility, and resorbability have been resolved, the latest biomimetic materials have been developed to meet the remaining requirements: osteoconduction, osteoinduction, and the ability to promote self-healing [105, 106]. Third-generation biomaterials, bioactive glasses, and macroporous foams with molecular modifications provide scaffolding for osteoconduction and growth factors for osteoinduction, thereby stimulating bone regeneration [105].

## Summary

Biomimetic materials and 3D printing have been employed to improve surgical and treatment outcomes. Biomimetic materials that imitate natural structures and biological properties are often used to design complicated nanoscale scaffold structures [107]. 3D printing technology is crucial for the successful and precise construction of biomimetic materials. Scaffolds imitating 3D porous structures similar to those of native bone have recently been manufactured using innovative 3D printing technology [108]. In 2018, Montalbano et al. developed 3D printed biomimetic scaffolds with hybrid bioactive material, consisting of type I collagen and strontium-containing mesoporous bioactive glasses, to facilitate osteogenesis [107]. Similarly, Main et al. invented a personalized customized implant composed of mineralized collagen (MC), which was used to repair large-scale weight-bearing bone defects [83].

The role of biomimetic materials and 3D printing in medicine is now widely recognized by medical experts. The scope of application covers several medical aspects, such as preoperative simulation, patient education, surgical training, intraoperative navigation, and the development of surgical tools, orthopedic implants, anatomic models, and patient-specific implantable materials in diverse surgical fields, including but not limited to orthopedics, maxillofacial surgery, cranial surgery, and spinal surgery [84, 86, 88, 89, 95]. Above all, the prognosis of patients with OVCFs is expected to significantly improve with developments in these technologies.

## Multimodal therapy

Multimodal therapy refers to the combination of several treatment approaches based on the patient’s specific condition and type of pathology. Clinicians widely use multimodal therapy in patients with middle- and late-stage malignant tumors to increase the cure rate, prolong the survival period, and improve the quality of life. Related treatments include surgical resection, chemotherapy, radiotherapy, and the emerging tumor-treating fields (TT fields) therapy [109, 110]. Several reports have shown that multimodal therapy can largely alleviate malignant tumors, such as metastatic penile cancer and advanced penile squamous cell carcinoma (pSCC), or even kill surviving tumors [111–113].

Similarly, accumulating evidence suggests that multimodal therapy could be effective in treating chronic metabolic diseases, such as obesity and diabetes. For instance, the combination of lifestyle changes, medication, and bariatric surgery has been shown help patients with obesity lose weight [114, 115]. Similarly, lifestyle changes and drug therapy can help treat diabetes [116]. In diabetic foot, surgical debridement and vascular reconstruction have also become part of multimodal therapy [117].

Similar to diabetes, osteoporosis is a chronic metabolic disease with a high incidence and severe complications. Therefore, multimodal therapies comprising MSC transplantation, exosome-based drug delivery systems, biomimetic materials, and 3D printing could be used in treating osteoporosis. However, there are limited studies that have investigated this approach. Stanco et al. inserted adipose-derived stem cells (ASCs) into a specific bio-ink and 3D-bioprinted them into multi-layered square-grid matrices to form tendon tissue [118]. Jang et al. developed bioinspired exosome-mimetic nanovesicles that maintain the intercellular communication function of exosomes with higher production yield and natural targeting capability to deliver chemotherapeutics [119]. Additionally, Narayanan et al. indicated that exosomes, which can induce lineage-specific differentiation of naive MSCs and bind to matrix proteins to anchor them to biomaterials, possess considerable potential in bone regenerative medicine [120]. This discovery suggests a new direction for future research on novel treatments for osteoporosis.

## Conclusion and prospects

Osteoporosis and OVCFs constitute severe health challenges, especially among older adults and postmenopausal individuals [11]. Traditional conservative therapies and present recommended therapies either only treat the symptoms without addressing the root cause of the disease, or present an increased risk of complications.

The development of MSC transplantation, exosome transplantation, biomimetic materials, and 3D printing technology has provided broad prospects for improving the treatment of osteoporosis and osteoporotic fractures. Despite the current difficulties in cell homing and regulation of the immunological response, a systemic or partial injection of MSCs can effectively remediate any cell deficiency caused by aging-related MSC apoptosis and adipogenic differentiation. Similarly, exosome transplantation plays a significant role in the regulation of signaling pathways and differentiation directions yet is associated with a high demand for experimental equipment and skilled researchers. The characteristics of biomimetic materials and 3D printing contribute to the organic combination of these two technologies. This method has great potential in promoting bone regeneration, even though improvements are required in terms of the raw materials and structure design. Ample experimental results show that the application of MSCs, exosomes, biomimetic materials, and 3D printing has restored the balance between bone resorption and bone formation in various animal models of osteoporosis, thus demonstrating the great clinical transformation potential of these three modalities in osteoporosis treatment. Moreover, the application scope of biomaterials and 3D printing can be extended to the field of surgery assistance, medical training, and doctor-patient communication.

Although considerable advances have been made towards applying these technologies in osteoporosis treatment, future studies should examine the efficacy of multimodal therapy using these technologies for the treatment of osteoporosis.

## Data Availability

Not applicable.

## Abbreviations

ADSCs: Adipose-derived stem cells
AM: Additive manufacturing
ASCs: Adipose-derived stem cells
BMD: Bone mineral density
BMMs: Bone marrow-derived macrophages
BMMSCs: Bone marrow mesenchymal stem cells
BKP: Balloon kyphoplasty
BMSC: Bone marrow stromal cell
CFU-F: Fibroblast colony-forming units
CT or MR images: Computed tomography or magnetic resonance images
CXCR4: C-X-C motif chemokine receptor 4
DCs: Dendritic cells
EC: Endothelial cell
ESCs: Embryonic stem cells
DPSCs: Dental pulp stem cells
EPC: Endothelial progenitor cell
ESEs: Early sorting endosomes
HGF: Hepatocyte growth factor
hiPSC-MSC-Exos: Exosomes of MSCs derived from human induced pluripotent stem cells
IAC: Immunoaffinity capture
ILVs: Intraluminal vesicles
LSEs: Late sorting endosomes
MC: Mineralized collagen
MHC: Major histocompatibility complex
miRNA: Micro-RNA
MOB: Mineralizing osteoblasts
MSCs: Mesenchymal stem cells
MVBs: Multivesicular bodies
NK cells: Natural killer cells
OE: Olfactory epithelium
OVCFs: Osteoporotic vertebral compression fractures
OVX rats: Ovariectomized rats
PBF: Powder bed fusion
PPARγ: Peroxisome proliferation-activated receptor γ
pSCC: Penile squamous cell carcinoma
PVP: Percutaneous vertebroplasty
RANKL: Receptor activator of NF-κB ligand
Runx2: Runt-related transcription factor 2
Ta: Tantalum
Ti: Titanium
TNF: Tumor necrosis factor
TT fields: Tumor-treating fields
UCMSCs: Umbilical cord MSCs
2D projections: 2-Dimensional projections
